# Insights into Intramuscular Connective Tissue Associated with Wooden Breast Myopathy in Fast-Growing Broiler Chickens

**DOI:** 10.3390/foods12122375

**Published:** 2023-06-15

**Authors:** Yulong Zhang, Mingyuan Huang, Xuefei Shao, Feiyu Zhang, Zhen Li, Yun Bai, Xinglian Xu, Peng Wang, Tinghui Zhao

**Affiliations:** 1 College of Food Science and Technology, Nanjing Agricultural University, Nanjing 210095, Chinaxlxus@njau.edu.cn (X.X.); 2 Jiangsu Synergetic Innovation Center of Meat Production and Processing, Nanjing 210095, China; 3 National Center of Meat Quality and Safety Control, Nanjing 210095, China; 4 Ninglang Animal Husbandry Work Instructing Station, Lijiang 674301, China

**Keywords:** *pectoralis major*, wooden breast myopathy, connective tissue, collagen, physicochemical property

## Abstract

Wooden breast myopathy (WBM) is a meat abnormality affecting *pectoralis majors* (PMs) of fast-growing broiler chickens. WBM-affected PMs exhibited varied meat qualities with increasing WBM severity. Normal PMs (NOR), mild WBM-affected PMs (MIL), moderate WBM-affected PMs (MOD), and severe WBM-affected PMs (SEV) were selected as raw materials. The structure and organization of connective tissue and fibrillar collagen were investigated through immersing with sodium hydroxide solution, Masson trichrome staining, and using an electron microscope. The mechanical strength of intramuscular connective tissue was analyzed via the shear force of samples treated with sodium hydroxide solution. The thermal property and secondary structure of connective tissue were analyzed by differential scanning calorimetry and Fourier transform infrared spectroscopy. The obtained connective tissue was dissolved in a sodium hydroxide solution for the evaluation of the physicochemical properties of proteins, including particle size, molecular weight, surface hydrophobicity, and intrinsic fluorescence. In particular, the particle size was measured using a zeta potential instrument. The molecular weight was analyzed by sodium dodecyl sulfate polyacrylamide gel electrophoresis. The surface hydrophobicity and intrinsic fluorescence were measured by spectroscopy technology. Histologically, macrophage infiltration, myodegeneration and necrosis, regeneration, fibrous connective tissue, and thickened perimysial connective tissue were observed in WBM-affected PMs, especially SEV with fibrosis, including blood vessels. Compared with NOR, WBM led to increased average diameter of the collagen fibrils in perimysial (36.61 nm of NOR to 69.73 nm of SEV) and endomysial (34.19 nm of NOR to 56.93 nm of SEV) layers. A significant increase (*p* < 0.05) was observed in the mechanical strength (2.05 N to 5.55 N) of fresh PMs and the thermal transition temperature (onset temperature (T_O_), 61.53 °C to 67.50 °C; maximum transition temperature (T_M_), 66.46 °C to 70.18 °C; termination temperature (T_E_), 77.20 °C to 80.88 °C) of connective tissue from NOR to SEV. Cooking decreased the mechanical strength, and MOD samples showed the highest mechanical strength (1.24 N, *p* < 0.05), followed by SEV (0.96 N), MIL (0.93 N), and NOR (0.72 N). For proteins in connective tissue, random coil (19.64% to 29.61%, *p* < 0.0001), particle size (*p* < 0.05), and surface hydrophobicity (*p* < 0.05) increased with the decrease in the α-helix (14.61% to 11.54%, *p* < 0.0001), β-sheet (45.71% to 32.80%, *p* < 0.0001), and intrinsic fluorescence of proteins from NOR to SEV. The molecular weights of intramuscular connective tissue proteins were in the ranges of >270 kDa, 180–270 kDa, 110–180 kDa, 95–100 kDa, and <15 kDa. Taken together, WBM resulted in thickened organization, tightly packed collagen fibrils, increased mechanical strength and thermal temperature, and increased particle size, surface hydrophobicity, and intrinsic fluorescence of proteins in connective tissue, as the WBM severity increased.

## 1. Introduction

Genetic selection meets the growth requirement for broiler chickens and the increasing demand for chicken meat, but this trend has resulted in emerging diseases in broiler chickens—white striping myopathy [1], pale, soft, and exudative myopathy [2], and wooden breast myopathy (WBM) [3]. These diseases reduce the meat quality owing to the altered visual appearance, water-holding capacity, chemical composition, and texture [4,5,6]. The incidence rate of WBM is in the range of 30–70% in China, with variations in different months, leading to a major hazard of WBM in the poultry industry.

The occurrence of WBM heavily affects the meat quality of *pectoralis majors* (PMs) from broiler chickens. Morphologically, WBM leads to changes in size, shape, yield, weight, thickness, and hardness of PMs [7]. These morphometric parameters increase with the severity of WBM, confirming the correlation between the occurrence of WBM and heavier and thicker PMs. WBM-affected PMs show diffused, harder, pale, and/or bulging areas, with/without white stripping, small petechial hemorrhages, and other characteristics [4]. Histologically, WBM-affected PMs show pathological tissue that reveals macrophage infiltration, myodegeneration and necrosis, regeneration, fibrous connective tissue, thickened perimysial connective tissue, extracellular collagen deposition, vacuolar and floccular degeneration, and fiber variability in size and shape [8,9]. This muscular abnormality has substantial implications on nutritional and technological qualities. WBM-affected PMs also have higher moisture, crude fat, and collagen; lower total protein; worse water-holding capacity; and altered texture properties [5,7]. Processed products (meatball, meat patty, sausage, and nugget) exhibit altered water-holding capacity and texture properties [10,11,12]. These alterations of PMs, resulting from muscular abnormalities, in the appearance, nutritional and technological qualities, consumer acceptance, and purchase intent, lead to the economic loss of the poultry industry.

WBM has been commonly observed in the PMs of fast-growing broiler chickens. The distinguishing macroscopical traits of WBM-affected PMs are the remarkable palpatory hardness in myopathic areas and the pale color, as well as the presence of bulges, a slimy surface, and small petechial hemorrhages [13]. The morphological structure of WBM-affected PMs exhibits inflammatory cell accumulation; muscle fiber necrosis and fibrosis; diffuse interstitial thickening with variable amounts of loose connective tissue, granulation tissue, and adipocytes; and complete reorganization of skeletal muscle structure [3,14]. The WBM severity varies based on the morphological structure of the fibrotic collagen and the extent of fibrosis [8,15]. Therefore, uncovering the properties of intramuscular connective tissue in WBM-affected PMs is essential. WBM-affected PMs present increased connective tissue, fibrosis of muscle fiber, and other abnormalities [16].

The epimysium, perimysium, and endomysium belong to muscle connective tissue. The epimysium, which consists of thick collagen bundles, covers the whole muscle. The perimysium consists of collagen fibrils surrounding bundles of muscle fibers. The endomysium surrounds individual muscle fibers. The organization and architecture of these three types of connective tissue determine the force transmission and impart mechanical properties to the muscle [17,18]. The vascular system in the connective tissue, in the form of vascular walls, and nerve walls consist of collagen, and they also determine the force transmission of muscle cells. Altered architecture of perimysial and endomysial connective tissues can affect muscle function and meat quality [19]. The structure and function of collagen fibrils and the amount of collagen protein are affected by mutations or disease, and changes in them influence cell–collagen interactions and tissue properties.

Our previous studies have shown the influences of WBM on the structure, proximate chemical compositions, collagen, texture, and oral property (masseter activity, chewing duration, chewing frequency, and particle size of meat bolus after chewing) of PMs, and the changes in connective tissue obviously affect meat quality [7,20]. Further studies are essential to investigate the influence of WBM on connective tissue. In this study, the correlations between WBM severity and the structure and organization of fibrillar collagen and connective tissue proteins were analyzed. Varied WBM-affected PMs were utilized as raw materials. Electron microscopy was used to comprehensively study the structure and organization of fibrillar collagen, and the physical properties of intramuscular connective tissue were evaluated via mechanical strength analysis, thermal transition, and Fourier transform infrared (FT-IR) spectroscopy. Meanwhile, the physicochemical properties of intramuscular connective tissue proteins, including particle size and molecular weight distribution, surface hydrophobicity, and intrinsic fluorescence, were assessed.

## 2. Materials and Methods

### 2.1. Broiler Chicken Breast Fillets

The average market age of Arbor Acres broiler chickens is 43 days (average weight: 2.68 kg). Broiler chickens were slaughtered through a commercial slaughter process, which consisted of electrical stunning at 120 mA for 15 s; bleeding for 240–300 s; scalding at 58–62 °C for 80 s; evisceration; chilling via water chilling at the first stage (<12 °C), second stage (<8 °C), and last stage (<4 °C, until reaching lower than 7 °C); and deboning. PMs of broiler chickens were selected for each experimental group from a deboning line (approximately 2–3 h postmortem) at a local processing plant (Yike Inc., Suqian, China). The PMs were assessed as WBM-unaffected (normal PMs, NOR, PMs that were flexible throughout), mild WBM-affected (MIL, PMs that were hard mainly in the cranial region but flexible otherwise), moderate WBM-affected (MOD, PMs that were hard throughout but flexible in mid to caudal region), and severe WBM-affected (SEV, PMs that were extremely hard and rigid throughout from cranial region to caudal tip) PMs by palpation in accordance with previously described criteria [20]. A total of 454 PMs were selected from the deboning line, including 124 NOR PMs, 103 MIL PMs, 117 MOD PMs, and 110 SEV PMs. In order to eliminate human error, all PMs were compressed via a texture analyzer. The test speed of the test probe was 1 mm/s, with a pre-test speed of 3 mm/s and a post-test speed of 5 mm/s. The maximum force required to compress 30% of the initial height of the sample was used as stress and expressed in Newtons (N). All fresh PMs were reclassified into four categories (NOR: cranial section stress < 11.44 N, middle section stress < 4.23 N, caudal section stress < 1.76 N; MIL: cranial section stress 11.44–15.78 N, middle section stress 4.23–5.31 N, caudal section stress 1.76–3.51 N; MOD: cranial section stress 15.78–21.35 N, middle section stress 5.31–10.85 N, caudal section stress 3.51–6.16 N; and SEV: cranial section stress > 21.35 N, middle section stress > 10.85 N, caudal section stress > 6.16 N) in accordance with a previously described 3D model [7].

All PMs were used in this study. In each group, 24 PMs were used to observe the microstructure of the connective tissue. That is, 24 PMs in each group were used to measure mechanical strength (24 raw PMs, 24 cooked PMs), with 24 PMs in each group for thermal property and 24 PMs in each group for secondary structure, particle size and molecular weight distribution, surface hydrophobicity, and intrinsic fluorescence. The experiment was repeated in four replicates, and five measurements at least were made for each repetition.

### 2.2. Extraction of Intramuscular Connective Tissue and Protein Solution Preparation

The extraction of intramuscular connective tissue was based on Wu’s method [21] with slight modification in consideration of meat sample type. PMs were cut into 10 × 10 × 5 mm^3^ pieces after the artificial removal of the epimysium by surgical blade. The muscle pieces (50 g) were mixed with 300 mL of cooled distilled water and homogenized in an ice bath at 4000 rpm for 10 s with an interval of 2 min for subsequent homogenization. The homogenate was filtered through a 100-mesh sieve to remove water and sarcoplasmic proteins. The residue was washed twice with 1.10 mol/L potassium chloride solution to obtain crude connective tissue. The crude connective tissue was stirred in 100 mL of potassium chloride solution (1 mol/L) for 24 h. The obtained crude connective tissue was then washed with 100 mL of sodium chloride solution (0.9%). It was finally washed twice using 100 mL of distilled water and centrifuged to obtain intramuscular connective tissue.

The connective tissue was cut into pieces, dissolved in 1 mol/L sodium hydroxide, stirred in a 4 °C room for 12 h, and continuously kept in the 4 °C room for 24 h. The protein content was determined using the biuret method, and the protein solution was adjusted and used for the analyses of particle size and molecular weight distribution, surface hydrophobicity, and intrinsic fluorescence.

### 2.3. Histological Evaluation

Histological analysis was performed using the procedure previously described by Dalgaard et al. [22]. Tissue samples in transverse sections were excised from the cranial region of 10 PMs from each WB category and immediately fixed in 4% paraformaldehyde in 0.1 mol/L phosphate buffer (pH 7.4) for 48 h. The specimens were then embedded in paraffin, and a 5 μm section cut longitudinal to the muscle fiber was stained using a Masson staining kit (Nanjing Jiancheng Bioengineering Institute, Nanjing, China) in accordance with the manufacturer’s instructions.

### 2.4. Transmission Electron Microscope (TEM) Observation

The microstructure of the endomysium in PMs was evaluated via a TEM [23,24,25]. TEM-sized pieces were excised from fresh PMs, fixed in 4% paraformaldehyde in 0.1 mol/L phosphate buffer (pH 7.4), and left overnight at 4 °C. The specimens were postfixed, stained with 1% osmium tetraoxide and 1% uranyl acetate for 1 h at room temperature, and dehydrated with 25%, 50%, 70%, and 90% absolute ethanol for 1 h. All samples were embedded in EMbed 812 Resin (Electron Microscopy Sciences, Hatfield, PA, USA). The specimens were examined and photographed via a Hitachi H-7650 TEM (Tokyo, Japan) with an accelerating voltage of 80.0 kV. The diameters of collagen and fibrils were measured using the software ImageJ 1.50i (National Institute of Health, Bethesda, MD, USA).

### 2.5. Mechanical Strength Measurement

Fresh PMs were placed into cooking bags and cooked in an 80 °C water bath until the core temperature of all PMs reached 76 °C. The mechanical strength of intramuscular connective tissue in PMs was measured with reference to Chang’s method [26]. Meat strips (10 × 10 × 5 mm^3^) from fresh and cooked PMs were fixed with 2.5% glutaraldehyde for 3 d and soaked in 10% sodium hydroxide solution for 5 d, followed by distilled water for 5 d. Connective samples were placed into channels (10 × 10 × 5 mm^3^) and fixed using 7.5% acrylamide solution (containing 1.5 mg/mL of ammonium persulfate) and 0.75 μL/mL tetramethylenediamine. Fixed samples were inoculated for 3 h. After the inoculation, the mechanical strength of intramuscular connective tissue was measured using a Texture Analyzer (Model TA.XT plus, Texture Technologies Corp., Hamilton, MA, USA) with a 50 kg load cell and an HDP/BSW probe. The test parameters were as follows: pre-speed, 2.00 mm/s; test speed, 1.00 mm/s; post-speed, 2.00 mm/s; and trigger force, 0.049 N.

### 2.6. Differential Scanning Calorimetry (DSC) Analysis

DSC was performed on a Perkin-Elmer DSC 8000 with reference to the method reported by Voutila et al. [27] after the calibration for temperature by using benzil and β-naphthol ethyl ether. The machine constant was obtained from the thermogram of a weighed amount of indium for its melting heat. The extracted intramuscular connective tissue above was cut into pieces. Approximately 15 mg of samples were sealed in Perkin-Elmer volatile sample pans, with the pan without any sample as the reference. Experiments were performed at a heating rate of 10 °C/min over the temperature range of 25–95 °C, after being kept at 25 °C for 2 min, with nitrogen as the carrier gas (2 mL/min). The peak area was measured and used to calculate the enthalpy of denaturation (ΔH).

### 2.7. FT-IR Spectroscopy

The amide III region, in combination with the amide I region, was used to investigate the secondary structure changes of intramuscular connective tissue from PMs with WBM severity. FT-IR spectra of intramuscular connective tissue in PMs were obtained via a Thermo-Nicolet iS10 FT-IR spectrometer (Nicolet Analytical Instruments, Madison, WI, USA) in accordance with Han’s method [28]. The infrared spectra from extracted intramuscular connective tissue were recorded between 400 and 4000 cm^−1^ with a resolution of 4 cm^−1^ and 32 scans. Fitting of the deconvoluted spectra was performed for the relative areas of the resolved amide I and III regions by using the peak analyzer software OMNIC 8.2 (Thermo Fisher Nicolet, Madison, WI, USA).

### 2.8. Particle Size and Molecular Weight Distribution of Connective Tissue Protein

Dynamic light scattering was performed to determine the particle size distribution of connective tissue proteins by using a Zetasizer Nano ZS 90 (Malvern Instrument Ltd., Great Malvern, UK) equipped with a 4 mW He–neon laser (λ = 633 nm) [29]. The solution (1 mg/mL) of proteins from connective tissue was placed in a 1 cm path length quartz cuvette. The particle size of proteins was determined with a detection angle of 90° at 25 °C. The autocorrelation function through the cumulant method was adopted to estimate the hydrodynamic diameters of connective tissue proteins on the basis of a single exponential fit of the autocorrelation function. According to the scattering intensity, the distributions of scattering particle size were monitored. The breadth of the size distribution was reflected by the polydispersity index value.

### 2.9. Sodium Dodecyl Sulfate Polyacrylamide Gel Electrophoresis (SDS-PAGE)

SDS-PAGE was used to determine the molecular weight distribution of connective tissue proteins. The concentration of the protein solution above (part 2.2) was adjusted to 4 mg/mL (pH 7.4), and the solution was mixed with 4× SDS loading buffer (1:3, *v*/*v*) and heated in a boiling water bath for 3–5 min. Protein samples were analyzed through a polyacrylamide electrophoresis gel (consisting of 5% stacking gel and 10% separating gel) on a Bio-Rad Mini Protean^®^ Tetra System (a cell electrophoresis apparatus) for one-dimensional gel electrophoresis by using Bio-Rad PowerPac^TM^ HC (a power supply), with a voltage of 120 V for the stacking gel and a voltage of 200 V for the separating gel. A broad range of prestained protein standards (Bio-Rad^®^, Hercules, CA, USA) were used. All gels were stained with Feto SDS-PAGE staining solution and then destained in distilled water. They were photographed via a ChemiDoc^TM^ MP imaging system and analyzed using Quantity One^®^ (Bio-Rad) software, version 4.6.3.

### 2.10. Measurement of Surface Hydrophobicity

Surface hydrophobicity was determined in accordance with the method of Zhang et al. [29]. The concentration of the protein solution above was adjusted to 1 mg/mL (pH 7.4), and 1 mL of the solution was mixed with 5 μL of 8-anilino-1-naphthalenesulfonic acid (ANS, 15 mmol/L in 0.1 mol/L phosphate buffer, pH 6.50) solution in darkness. The mixtures were kept at 25 °C for 20 min in darkness. After the full reaction of the mixtures, they were centrifuged at 4 °C and 10,000× *g* for 10 min to obtain the supernatant. The fluorescence intensity (FI) of the supernatant in each group was measured via a SpectraMax microplate reader (SpectraMax M^2e^, Molecular Devices, Sunnyvale, CA, USA) with a constant excitation wavelength of 355 nm, an emission wavelength in the range of 400–600 nm, integration every 1 s, and a step size of 5 nm.

### 2.11. Measurement of Intrinsic Fluorescence

Intrinsic fluorescence was performed according to Ding et al. [30], with a slight modification. The concentration of the protein solution above was adjusted to 0.5 mg/mL (pH 7.4). On the basis of the fixed settlement of the SpectraMax microplate reader (SpectraMax M^2e^, Molecular Devices, Sunnyvale, CA, USA) with the difference between the minimum settled emission wavelength and settled excitation wavelength ≥ 45 nm, the emission spectra of proteins (0.5 mg/mL) from connective tissue were recorded from 295 nm to 450 nm of emission wavelength with a constant excitation wavelength of 250 nm, integration every 1 s, and a step size of 1 nm, via the SpectraMax microplate reader.

### 2.12. Statistical Analysis

All statistical data were analyzed as a completely randomized design using the GLM procedure present in SAS software (SAS 8.2. SAS Inst. Inc., Raleigh, NC, USA). The ANOVA option of the GLM procedure present in SAS software was performed. Means were separated by Tukey’s HSD test of the GLM procedure. In all tests, *p* < 0.05 was considered significant. The SIMCA-14.1 software (UMETRICS, Umeå, Sweden) was used for orthogonal partial least squares discriminant analysis (OPLS-DA).

## 3. Results and Discussion

### 3.1. Collagen Organization

**Figure 1:**
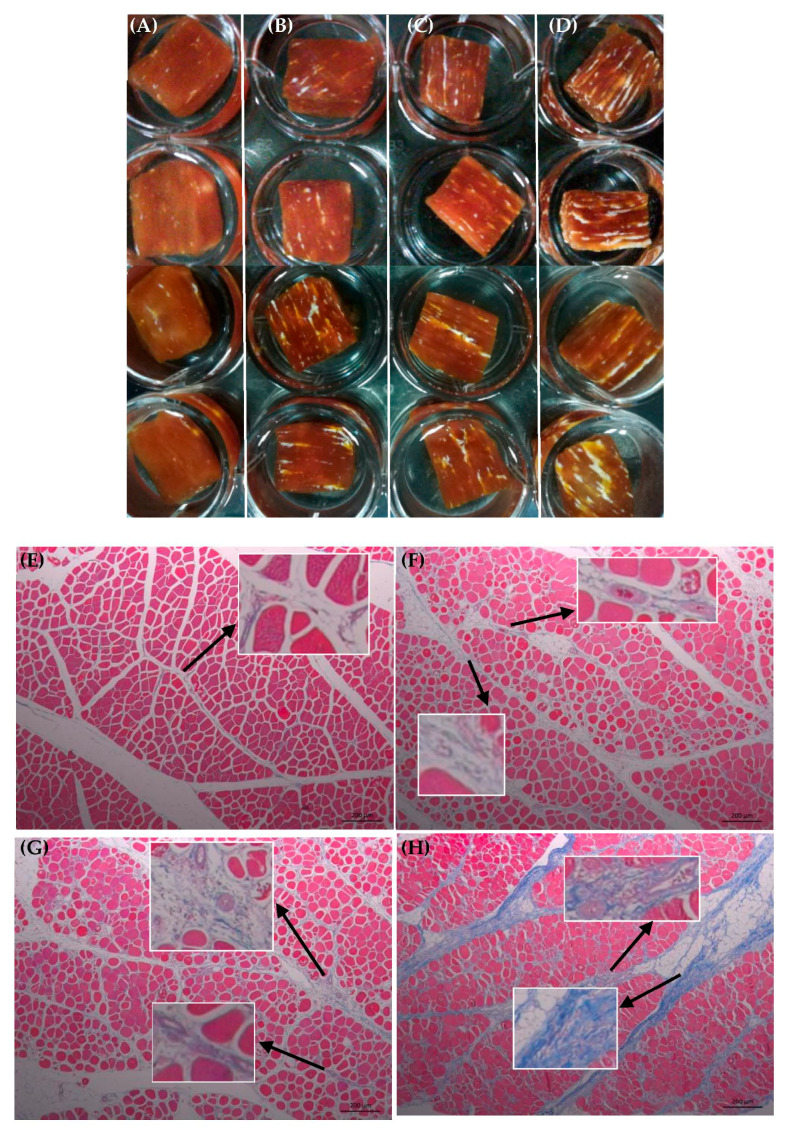
Sodium hydroxide soak (**A**–**D**) and Masson trichrome staining (**E**–**H**) of collagen organization in unaffected and WBM-affected PMs. (**A**,**E**) NOR, unaffected PMs; (**B**,**F**) MIL, mild WBM-affected PMs; (**C**,**G**) MOD, moderate WBM-affected PMs; (**D**,**H**) SEV, severe WBM-affected PMs. Scale bar = 200 μm (**E**–**H**).

The collagen organization was evaluated by sodium hydroxide soaking and Masson trichrome staining of unaffected and WBM-affected PMs (Figure 1). Figure 1A–D show an increase in connective tissue, especially SEV PMs. Owing to sodium hydroxide, the muscle fibers of all specimens from the PMs were partially dissolved, of which the specimens from the unaffected PMs had severely destroyed, softened, and swelled tissue. The amount of loose connective tissue was increased in WBM-affected PMs compared with that in unaffected ones (Figure 1A–D). The Masson trichrome staining showed abnormal muscle tissue in WBM-affected PMs that presented macrophage infiltration, polyphasic myodegeneration and necrosis, regeneration, proliferation and thickened loose connective tissue, adipocytes, and fiber variability in number, size, and shape. Blood vessels, lymphatic vessels, and nerves also contain collagen, and they can be stained blue. Via Masson trichrome staining, some vessels containing erythrocytes were considered blood vessels. However, whether some vessels in the tissue are blood vessels or lymphatic vessels is difficult to distinguish given their similarities. Fat tissue and blood vessels in connective tissue were also observed in WBM-affected PMs. Considering the size and structure of nerves, the specialist pathologists still did not distinguish nervous structure in these Masson trichrome staining photos. The increased blood vessels in WBM-affected PMs revealed small petechial hemorrhages on the surfaces of some WBM-affected PMs. Thickened perimysial layers were observed in WBM-affected PMs compared with unaffected PMs. Severe WBM-affected PMs had the greatest amount of connective tissue, indicating extensive fibrosis, which is similar to the findings of Velleman and Clark [15]. Some authors observed diffuse interstitial thickening with variable amounts of loose connective tissue, granulation tissue, and fibrosis in the WBM-affected areas [5], and thickened perimysial and endomysial layers in WBM-affected PMs [31]. PMs had increased connective tissue space, collagen deposition, myofiber necrosis, and inflammatory cell accumulation with WBM severity [5,7,9,13,14,15]. Specimens from WBM-affected PMs contained dense collagen fiber bundles that were randomly aligned. These alterations in the tissue of WBM-affected PMs not only increased collagen concentration, but may be associated with the phenotypic hardness of fresh WBM-affected PMs.

### 3.2. Collagen Fibrillar Structure

**Figure 2:**
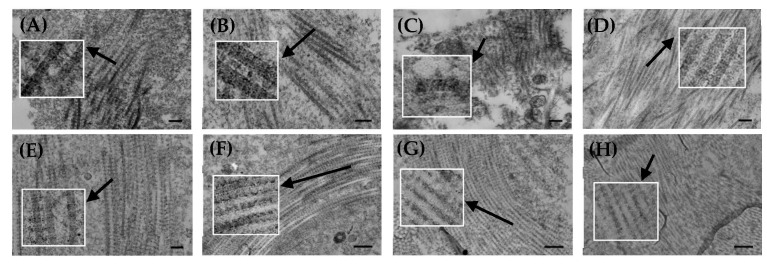
Electron microscope images of collagen fibrils in unaffected and WBM-affected PMs. (**A**–**D**) SEM of perimysium, scale bar = 200 nm (**A**,**B**) and 400 nm (**C**,**D**); (**E**–**H**) TEM of endomysium, scale bar = 200 nm (**E**), 400 nm (**G**), and 500 nm (**F**,**H**). (**A**,**E**) NOR, unaffected PMs; (**B**,**F**) MIL, mild WBM-affected PMs; (**C**,**G**) MOD, moderate WBM-affected PMs; (**D**,**H**) SEV, severe WBM-affected PMs. The boxes show enlarged images of the collagen fibrils.

Perimysial and endomysial layers were observed from the photos taken via a TEM. Figure 2 shows representative images of collagen fibrils from unaffected and WBM-affected specimens. Collagen fibrils were irregularly densely arranged and morphologically transformed, and these fibrils varied in density arrangement and size. Collagen fibrils from all PMs specimens exhibited the characteristic collagen fibril structure with light and dark bands. Clear electron-dense areas were observed in the perimysium and endomysium of unaffected PMs. In WBM-affected PMs, the fibrotic regions, perimysium, and endomysium contained the fibril-associated electron-dense material, and collagen fibrils were densely arranged. Similar results were found in a study performed by Velleman et al. [25]. Tonniges et al. [32] determined that WBM resulted in decreased thin collagen fibril bundles (<1.83 μm) and increased thick fibril bundles (>2.93 μm). Dense collagen fibrils were clearly observed in severe WBM-affected perimysium and endomysium. From the Masson trichrome staining in Figure 1, SEV PMs showed fibrosis. Fibrosis is a pathological buildup of the extracellular matrix, and leads to an increase in tissue stiffness [33]. Fibrotic muscle demonstrates increased collagen cross-linking [34], and highly cross-linked collagen is related to muscle stiffness [35]. The increased stiffness of WBM-affected PMs is likely attributed to the structural characteristics and extent of fibrotic collagen, except for the increased collagen content, and other tissues that contain collagen fibers. Alterations in the structural organization of collagen fibrils affect tissue structure and function [36], meat tenderness [37,38], the phenotypic hard consistency, and the independence of the other morphological features of WBM-affected PMs. Decorin directly binds to fibrillar collagen to affect the formation of hydroxylysylpyridinoline (HP) cross-linking and the collagen quarter-staggered array [39,40,41]. Increased HP cross-linking can stiffen muscle tissue, leading to a hard muscle. Decorin is highly expressed in WBM-affected PMs [15], and its increased expression results in a dense arrangement of collagen fibrils and an elevated level of collagen cross-linking. In the present study, the collagen fibrillar structure changed with the occurrence of WBM. The changes were clearly observable, especially in severe WBM-affected perimysium and endomysium, regardless of the cross-linking.

**Figure 3:**
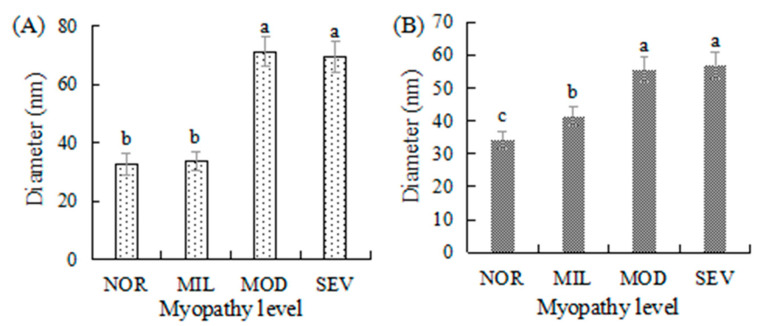
The average diameter of collagen fibrils. Different letters above columns show significant difference (*p* < 0.05). (**A**) perimysium, (**B**) endomysium. NOR, unaffected PMs; MIL, mild WBM-affected PMs; MOD, moderate WBM-affected PMs; SEV, severe WBM-affected PMs.

The average diameter of collagen fibrils from the perimysial and endomysial regions in unaffected and WBM-affected PMs is shown in Figure 3. The average diameters of perimysial and endomysial collagen fibrils from MOD and SEV PMs were larger than those from MIL and NOR PMs (*p* < 0.05). For the average diameter of perimysial collagen fibrils, there was no difference between the NOR and MIL specimens. The endomysial collagen fibrils in MIL PMs had an average diameter larger than those in NOR PMs (*p* < 0.05). The collagen fibrils in WBM-affected PMs individually varied in diameter. The above results in the current study are different from those described by Velleman et al. [25] because of the WBM severity. A defining phenotypic feature of WBM-affected PMs is the palpably hard consistency caused by the degrees of increased myofiber necrosis, immune cell infiltration, and fibrosis [3,5,15,22]. The increased collagen concentration does not affect the consistency of WBM-affected PMs because the collagen concentration in muscle does not correlate with the muscle stiffness, and only correlates with collagen organization, such as collagen fibril diameter, cross-linking, fibril alignment, and packing [42]. A previous study found that WBM-affected PMs had an increased number of tightly packed and aligned collagen fibers [15]. Collagen fibril diameter is associated with collagen cross-linking [43], and the cross-linking can enhance the stability of collagen fibers, and result in the formation of larger fibrils [44]. Highly expressed decorin in WBM-affected PMs promotes the formation of HP cross-linking, which links three collagen triple helixes and continues to link additional helixes to increase the fibril diameter.

### 3.3. Mechanical Strength

**Figure 4:**
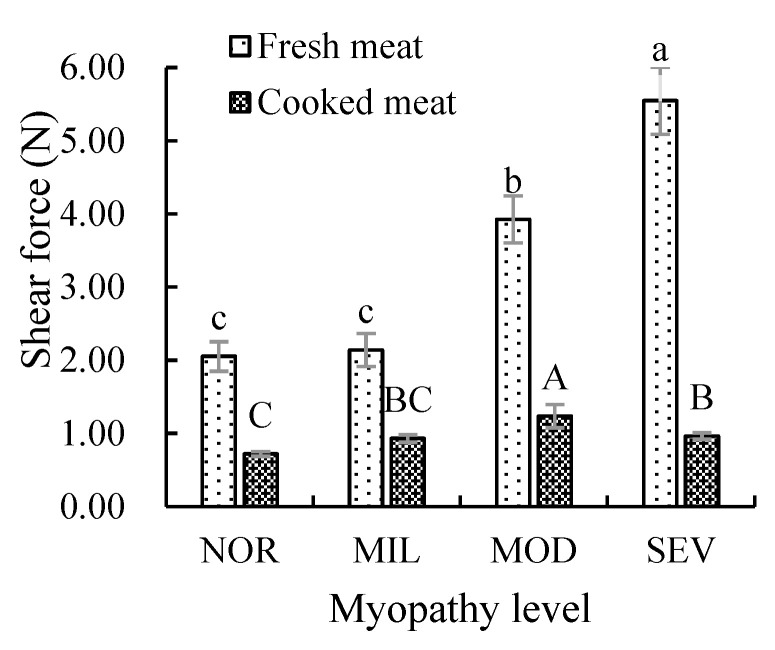
Mechanical strength of intramuscular connective tissue in chicken breast fillets. Different letters above columns show difference as to the same type of samples (*p* < 0.05). NOR, unaffected PMs; MIL, mild WBM-affected PMs; MOD, moderate WBM-affected PMs; SEV, severe WBM-affected PMs.

Intramuscular connective tissue, mainly including perimysium and endomysium, supports and connects various tissues and organs in the body, giving muscles certain elasticity and hardness. The mechanical strength of the intramuscular connective tissue of WBM-affected PMs before and after cooking is shown in Figure 4. With increasing WBM, the mechanical strength of intramuscular connective tissues in fresh PMs increased gradually, and the intramuscular connective tissues in SEV PMs had the significantly highest mechanical strength (*p* < 0.05), followed by those in MOD, MIL, and NOR PMs. WBM altered the tissue structure and composition of PMs [5], and the increased tissue hardness and firmness of WBM-affected PMs resulted from the accumulation of interstitial connective tissue, diffuse thickening in the interstitial fraction, and the deposition of variable amounts of loose connective tissue [15,25], high moisture, and myofibril swelling of muscle tissue, leading to an increase in the shear force of WBM-affected PMs [45]. A high degree of the inherent strength of connective tissue affected the resistance to shearing of muscle tissue. The structure and content of intermuscular connective tissue affect the mechanical strength of meat [14].

The mechanical strength of intramuscular connective tissue of PMs obviously decreased after cooking. The mechanical strength of intramuscular connective tissue of MOD PMs was significantly the highest (*p* < 0.05), followed by those of SEV and MIL PMs, and there was no significance between SEV and MIL (*p* > 0.05). This result is similar to the changes in shear force of cooked PMs with different WBM severity reported by Zhang et al. [7]. Cooking altered the fiber structure of muscle and collagen, and destroyed meat state. After cooking, MOD PMs maintained a tighter meat structure than SEV PMs. Due to fibrotic tissue, altered muscle fiber, and the large amount of fat tissues, the structure of SEV PMs was severely destroyed during cooking [7], consequently leading to altered intramuscular connective tissue and collagen. Destroyed meat tissue is easily attacked by sodium hydroxide to decrease shear force. The mechanical strength of SEV PMs was significantly higher than that of NOR PMs (*p* < 0.05). In the process of meat processing, the mechanical strength of muscle connective tissue depends on the arrangement and direction of individual collagen fibers and the effect of other noncollagen components, and it is related to the contraction of myofibrils to a certain extent [46]. The change in the mechanical strength of the connective tissue is related to protein structure and solubility, which reflects the change in meat tenderness (shear force) to a certain extent [47]. The contents of total collagen and insoluble collagen in PMs increased with the increase in the WBM degree [7]. However, the mechanical strength of the intramuscular connective tissue in PMs did not show a similar increase after cooking, and demonstrated a tendency to increase first and then decrease. Studies have reported a positive correlation between the content change of insoluble collagen and the mechanical strength of connective tissue [26,48,49]. SEV PMs had higher contents of total collagen and insoluble collagen, and a lower content of heat-soluble collagen, than MOD PMs [7]. The tissue structure of SEV PMs changed greatly, especially the myofibril and connective tissue. More connective tissues were exposed in the cooking process, which led to loose tissue structures caused by the thermal aggregation of collagen and other proteins in SEV PMs after cooking to reduce the shear force of the muscle.

### 3.4. Thermogram of the Connective Tissue

**Figure 5:**
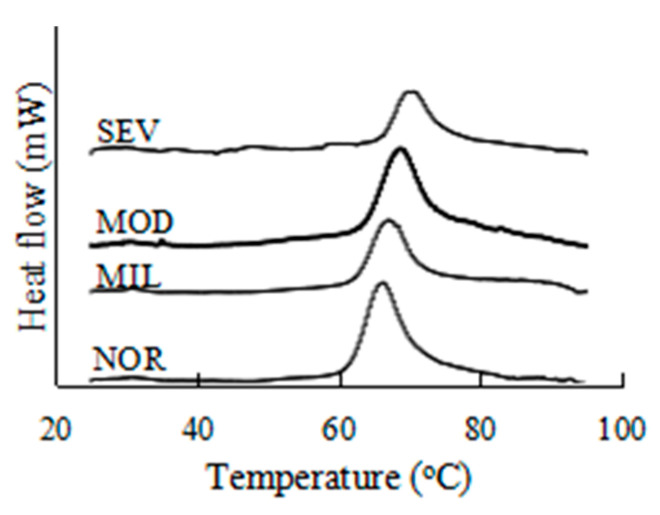
DSC heat flow diagram of intramuscular connective tissue. NOR, unaffected PMs; MIL, mild WBM-affected PMs; MOD, moderate WBM-affected PMs; SEV, severe WBM-affected PMs.

Thermograms of extracted connective tissues are illustrated in Figure 5. The thermograms of the intramuscular connective tissues from all PMs show an obvious peak, which indicates that the thermal transition temperatures of proteins in intramuscular connective tissues were similar. The thermal transition temperature of the intramuscular connective tissue of the PMs increased with the increase in the WBM degree. The thermal transition temperature of the connective tissue is related to the degree of structural destruction of proteins (mainly collagen) in the connective tissue [50]. The collagen in muscle tissues of livestock and poultry of different breeds or different feeding ages of the same breed dramatically varies in thermal stability, content, and degree of intermolecular and intramolecular cross-linking [51,52,53]. In muscle tissues of young livestock and poultry, collagen is not stable upon exposure to heat and acidity, and is susceptible to degradation caused by changes in collagenase, ionic strength, and temperature [54]. With the growth of livestock and poultry, the reducing cross-linking of collagen is transformed into a more stable nonreducing cross-linking, decreasing the sensitivity of connective tissue to heat and acid, and the thermal solubility of collagen decreases [55]. The increased thermal stability and mechanical strength of connective tissue are mainly related to the chemical properties and amino acid composition of collagen after intermolecular covalent cross-linking [56].

The denaturation temperature and denaturation enthalpy reflect the thermal stability of protein, in which high values indicate the high thermal stability of protein [57,58]. As shown in Table 1, WBM led to increased T_O_, T_M_, and T_E_ of the intramuscular connective tissue. The T_O_, T_M_, and T_E_ of the intramuscular connective tissue increased significantly from 61.53, 66.46, and 77.20 °C to 67.50, 70.18, and 80.88 °C (*p* < 0.05) as the WBM worsened. The maximum transition temperature of intramuscular connective tissue in unaffected PMs is close to the result reported by Kijowski et al. [59]. The trend of the thermal transition temperature of intramuscular connective tissue was similar to that of the mechanical strength of PMs before cooking. The enthalpy of connective tissues from WBM-affected PMs decreased to 2.25 J/g compared with that (4.95 J/g) of normal PMs. The difference in protein content and cross-linking in fresh connective tissue possibly resulted in the difference in denaturing enthalpy between connective tissues from WBM-unaffected and WBM-affected PMs. From Figure 1, increased fat tissue could be observed in the MIL, MOD, and SEV samples, resulting in increased fat and decreased collagen in the connective tissue. Studies have reported obvious compositional differences between WBM-unaffected and WBM-affected PMs, especially for increased total collagen and fat [4,5,7], but the results for the enthalpies clearly show decreasing amounts of proteins in connective tissues from WBM-affected PMs. Consequently, the difference in denaturing enthalpy between connective tissues from WBM-unaffected and WBM-affected PMs might be attributed to decreased protein content. The thermal denaturation temperature of proteins is believed to be the result of the long-term adaptation of organisms to their growing environment [60]. The thermal denaturation temperature of the connective tissue in WBM-affected PMs resulted from the alteration of structural properties and amino acid composition under the conditions of genetic selection and feeding increase. An increased thermal stability of connective tissue is also strongly related to the cross-linking of collagen, which can improve its thermal stability [61,62]. WBM-affected PMs contain substantial highly cross-linked collagen fibers [25], leading to increased phenotypic hardness and thermal stability of collagen and decreased tenderness of WBM-affected PMs.

### 3.5. Secondary Structure

**Figure 6:**
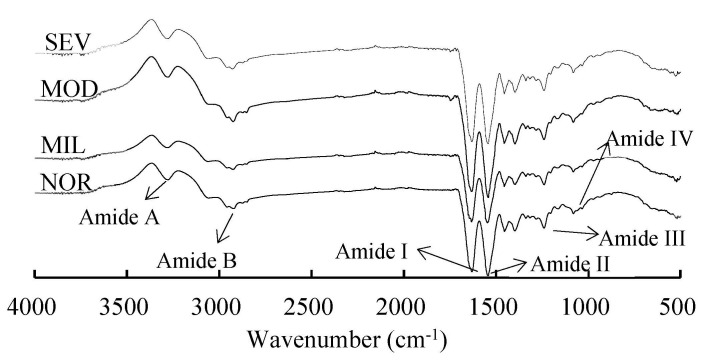
Fourier transform infrared (FT−IR) spectra of intramuscular connective tissue. NOR, unaffected PMs; MIL, mild WBM-affected PMs; MOD, moderate WBM-affected PMs; SEV, severe WBM-affected PMs.

The amide bond of protein has many distinct vibrational modes, mainly including amide A, amide B, amide I, amide II, amide III, and amide IV regions (Figure 6). The amide I region (1700–1600 cm^−1^) [63] and the amide III region (1330–1220 cm^−1^) [64] are usually used to investigate the secondary structure (α-helix, β-sheet, β-turn, and random coil) of proteins. The amide I region mostly originates from the C=O stretching vibration (80%) and from both N–H bending and C–N stretching vibrations (20%) [65]; however, interpretation of protein spectra in the amide I region is somewhat problematic because of the interference of O–H vibrations caused by liquid water or water vapor and overlapping peaks. Even though the amide II region has a relatively strong intensity, it is minimally sensitive to the secondary structure changes of proteins, and the bands in its region are strongly overlapped by bands originating from amino acid side chain vibrations [66]. The amide III region mainly originates from N–H bending vibration (30%) and C–N stretching vibration (30%), and from C–C stretching vibration (20%) and C–H bending vibration (10%) [65]. The signal of the amide III bands with no water interference is 5–10-fold weaker than that of the amide I bands, and the drawbacks of the amide I region caused by the overlapping of peaks are not found in this region [67]. In this study, the amide I and III bands were centered at 1632.81 and 1240.22 cm^−1^, respectively, for all intramuscular connective tissues from PMs, which is similar to the results for dried blood serum obtained by Titus et al. [68].

As shown in Table 2, there was a significant decrease in α-helix (from 14.61% to 11.54%) and β-sheet (from 45.71% to 32.80%) and a significant increase in β-turn (from 20.78% to 25.08%) and random coil (from 19.64% to 29.61%) with increasing WBM severity (*p* < 0.05). The secondary structure of intramuscular connective tissue from NOR PMs was mainly β-sheet, followed by β-turn and random coil. The increased random coil and the decreased β-turn indicates the unfolding and increased disorder of collagen molecules [69]. The TGase-catalyzed cross-linking reaction changes the secondary structure of proteins from α-helix and unordered structures to β-sheet and β-turn [70]. WBM caused increased cross-linking of intramuscular connective tissue, resulting in changes in the secondary structure of its proteins. The WBM occurrence altered the components of the extracellular matrix and types of collagen and collagen fragments, as well as changed the contents of total collagen and soluble collagen [7], resulting in an altered secondary structure.

### 3.6. Particle Size

**Figure 7:**
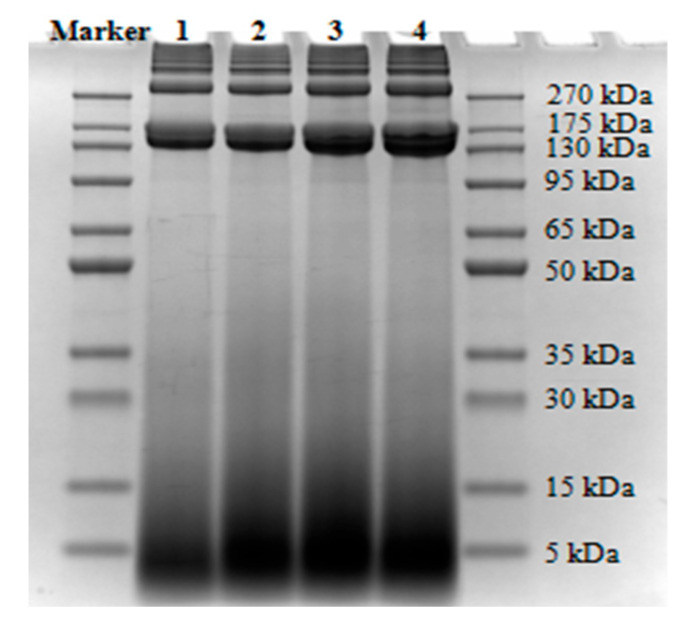
Molecular weight distribution of intramuscular connective tissue proteins from PMs. Lane 1, NOR; lane 2, MIL; lane 3, MOD; lane 4, SEV. NOR, unaffected PMs; MIL, mild WBM-affected PMs; MOD, moderate WBM-affected PMs; SEV, severe WBM-affected PMs.

Figure 7 shows the molecular weight distribution of intramuscular connective tissue proteins from PMs. Compared with that of unaffected PMs (1322.05 nm), the average particle size of intramuscular connective tissue proteins from WBM-affected PMs increased. The particle size of connective tissue proteins is affected by protein source, extraction method, solvent, molecular weight, and the interaction between proteins. A previous study reported a particle size of 422 nm for acid-soluble collagen from a soft-shelled turtle and 338 nm for acid-soluble collagen extracted via ultrasound [71]. The particle size of chicken lung collagen extracted by the acid method was 340 nm, whereas that of collagen extracted by the ultrasound-assisted acid method was just 295 nm [72]. The WBM occurrence causes increased heat-soluble collagen and total collagen in PMs [7]. WBM-affected PMs contain a large number of highly cross-linked collagen fibers [25]. The highly cross-linked and soluble collagen concentration affects the solubility of intramuscular connective tissue proteins from WBM-affected PMs, resulting in alterations in protein particle size. In consideration of molecular weight distribution, the electrophoretic bands of connective tissue proteins from WBM-affected PMs were similar to those from unaffected PMs, indicating that the profiles of connective tissue proteins were basically similar. As the WBM worsened, the width of bands of proteins increased. The ranges of the molecular weight of intramuscular connective tissue proteins include >270 kDa, 180–270 kDa, 110–180 kDa, 95–100 kDa, and <15 kDa. Under a strong alkaline condition, a fraction of the connective tissue proteins were dissolved because of the destruction of the connective tissue network, and degraded with increasing dissolution time, which resulted in the increased solubility and low molecular weight of the connective tissue proteins.

### 3.7. Surface Hydrophobicity

**Figure 8:**
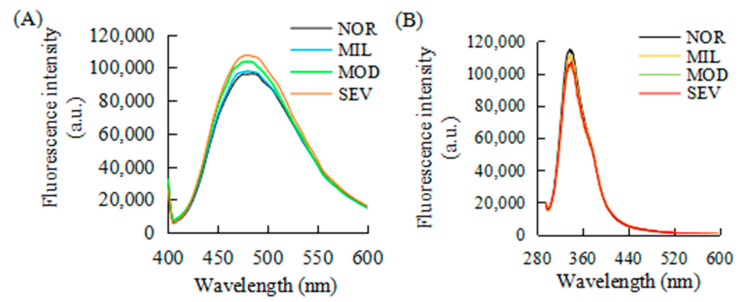
Surface hydrophobicity (**A**) and intrinsic fluorescence (**B**) of intramuscular connective tissue proteins. NOR, unaffected PMs; MIL, mild WBM-affected PMs; MOD, moderate WBM-affected PMs; SEV, severe WBM-affected PMs.

The surface hydrophobicity of proteins is the most important force required to maintain the tertiary structure of proteins [73], affecting the structural stability, conformation, and functional properties (such as foaming capacity and emulsifying capacity) of proteins [74]. The content of hydrophobic amino acid residues exposed by a protein is often used to indicate the hydrophobicity of a protein [75], and the optimal balance of hydrophobic and hydrophilic residues is conducive to the optimal function of a protein [76]. The surface hydrophobicity of connective tissue proteins is shown in Figure 8A. The maximum fluorescence emission wavelength of connective tissue proteins was 480 nm, which was different from the maximum absorption wavelength of myosin of 460 nm [77]. These differences might result from the differences in protein type, state, and solution environment. The fluorescence intensities of connective tissue proteins from NOR and MIL PMs were very close, and significantly lower than those of connective tissue proteins from MOD and SEV PMs. The FI of connective tissue proteins was the highest in SEV PMs. Li and Jiang [78] found that the surface hydrophobicity of soybean 7S and 11S proteins was positively correlated with the total amount of hydrophobic and hydrophilic amino acids. The main hydrophobic amino acids are glycine, valine, alanine, leucine, isoleucine, and others. These amino acids maintain the tertiary structure of a protein through hydrophobic interaction. As a main component of connective tissue, collagen contains a certain amount of hydrophobic amino acids, and their changes affect the functional properties [79]. The WBM occurrence may change the protein composition of connective tissue, resulting in an increased content of hydrophobic amino acid residues of proteins and increased hydrophobic groups on the protein surface, ultimately leading to increased hydrophobicity on the protein surface. The molecular state of connective tissue proteins also affects the exposure of hydrophobic groups. Hydrophobicity is closely related to the spatial conformation of protein molecules and the exposure degree of hydrophobic residues [80]. When proteins are folded and aggregated, hydrophobic groups are embedded in the inner region of the proteins and surrounded by a nonpolar environment. The number of ANS molecules binding to the nonpolar region of the protein decreases; consequently, the surface hydrophobicity of the protein decreases. When protein molecules stretch and unfold, hydrophobic groups are exposed to aqueous solution, which increases the probability of ANS molecules binding with hydrophobic regions, and thus increases the surface hydrophobicity of proteins [81].

### 3.8. Intrinsic Fluorescence

The intrinsic fluorescence of a protein is derived from tyrosine, tryptophan, and phenylalanine, and directly reflects the properties of these amino acid residues in proteins and the changes in their surrounding microenvironment [82]. As shown in Figure 8B, the connective tissue protein had a characteristic peak at 338 nm with an excitation wavelength of 250 nm. This result is different from the maximum fluorescence emission wavelength of type I collagen of rat lung, which was reported to have a maximum emission wavelength of 364 nm with an excitation wavelength of 269 nm [83]. The maximum emission wavelength of type I collagen from calf skin was 306 nm with an excitation wavelength of 275 nm, and the addition of sericin from silk caused an obvious shift toward the longer wavelength side [84]. Many studies have shown that extracted collagen mainly contains alanine, glycine, proline, and hydroxyproline, with a certain amount of tyrosine and a small amount of phenylalanine [85,86]. Previous studies have reported that the intrinsic fluorescence of collagen is derived from phenylalanine and tyrosine [87,88]. Connective tissue contains elastin, reticulin, glycoprotein, and proteoglycan, not just the main collagen. The complex protein composition causes the maximum emission wavelength of intrinsic fluorescence to shift to a certain extent. Connective tissue proteins from NOR PMs had the highest FI, followed by those from MIL PMs. By contrast, connective tissue proteins from MOD and SEV PMs had the lowest FI. The difference in FI above is possibly related to protein composition and protein molecular state. The contraction of the side chain of a protein molecule causes decreased spaces of the adjacent amino acid side chain residues, which leads to enhanced FI and interaction between these residues. The stretch of a protein molecule resulted in an increased distance between the adjacent residues of specific amino acid side chain of the same molecule; consequently, it weakened the interaction between specific amino acid residues and FI [89]. The property of a protein is also related to its particle size. The formation of substantial protein aggregates locates the particle size of proteins in the large particle size range, and the specific amino acid residues of proteins are wrapped, which is responsible for the decreased exposure of these side chain residues and FI of proteins [80]. With the increase in protein particle size, the intrinsic FI of protein decreased. In connective tissue, the pyridine nuclear cross-linking structure of elastin makes elastin display fluorescence properties [90].

### 3.9. OPLS-DA Model

**Figure 9:**
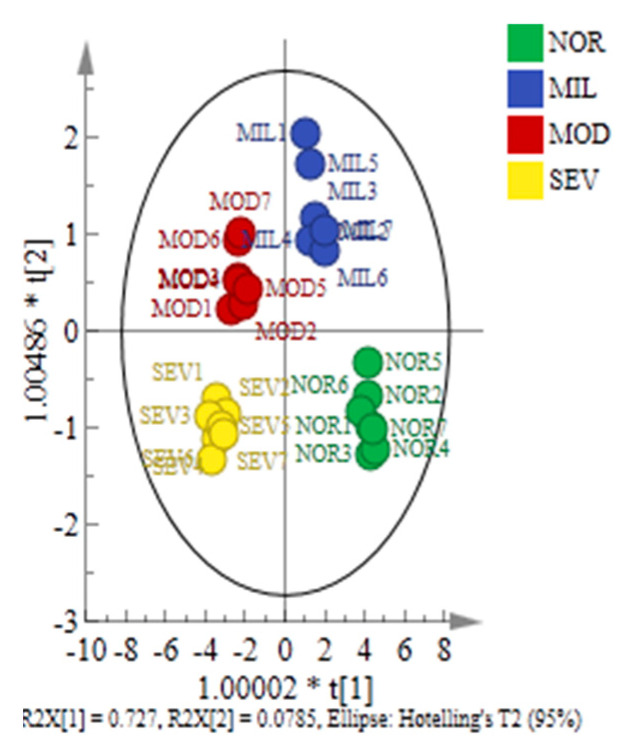
Score plot of OPLS-DA model. NOR, unaffected PMs; MIL, mild WBM-affected PMs; MOD, moderate WBM-affected PMs; SEV, severe WBM-affected PMs.

Through the OPLS-DA model, the intramuscular connective tissues from PMs were divided into four categories: NOR, located in the fourth quadrant; MIL, located in the first quadrant; MOD, located in the second quadrant; SEV, located in the third quadrant (Figure 9). The results above indicate intragroup and intergroup differences, suggesting that the WBM severity affected the structure and organization of fibrillar collagen and physicochemical properties of connective tissue proteins. The fitting coefficient of the independent variable R^2^X was 0.928, the fitting coefficient of the dependent variable R^2^Y was 0.896, and the predictive capacity Q^2^ was 0.859.

## 4. Conclusions

The WBM severity affect aspects of intramuscular connective tissue, especially physical attributes, such as mechanical strength, thermal property, and spectral characteristics. The occurrence of WBM led to changes in collagen fibrillar structure and collagen organization, and increased the mechanical strength, thermal stability, and secondary structure alteration of intramuscular connective tissue with WBM severity. Intramuscular connective tissue proteins exhibited increased particle size and surface hydrophobicity and decreased intrinsic FI. In sum, the occurrence of WBM altered the organization of connective tissues, secondary structure of proteins, molecular weight distribution, and intrinsic fluorescence, as well as increased mechanical strength, thermal temperature, and surface hydrophobicity.

Although the investigation of connective tissue from WBM-affected PMs was performed in this study, further research is essential to reveal the formation mechanisms of WBM. Future research will focus on signals that are related to fat tissue, connective tissue, the composition and cross-linking of collagen fibrils, and the binding of decorin to decrease the occurrence rate of WBM-affected PMs and improve meat quality.

## Figures and Tables

**Table 1:** Maximum transition temperature and enthalpy (ΔH) of fresh connective tissue.

Severity	T_O_ (°C)	T_M_ (°C)	T_E_ (°C)	ΔH (J/g)
NOR	61.53 ^c^	66.46 ^c^	77.20 ^b^	4.95 ^a^
MIL	63.40 ^b^	67.19 ^bc^	77.62 ^b^	2.41 ^b^
MOD	64.66 ^b^	68.35 ^b^	79.25 ^a^	2.34 ^b^
SEV	67.50 ^a^	70.18 ^a^	80.88 ^a^	2.25 ^b^
s.e.m.	0.24	0.60	0.37	0.42
*p* value	<0.0001	<0.0001	<0.0001	<0.0001

Different letters in same column show significant difference (*p* < 0.05). s.e.m. = standard error of mean. NOR, unaffected PMs; MIL, mild WBM-affected PMs; MOD, moderate WBM-affected PMs; SEV, severe WBM-affected PMs. T_O_, onset temperature; T_M_, maximum temperature; T_E_, termination temperature.

**Table 2:** Relative content of protein secondary structures of connective tissue.

Myopathy Level	α-Helix (%)	β-Sheet (%)	β-Turn (%)	Random Coil (%)
NOR	14.61 ^a^	45.71 ^a^	20.78 ^c^	19.64 ^b^
MIL	12.98 ^b^	41.17 ^b^	24.64 ^ab^	21.24 ^b^
MOD	11.64 ^c^	36.40 ^c^	23.24 ^b^	28.67 ^a^
SEV	11.54 ^c^	32.80 ^d^	25.08 ^a^	29.61 ^a^
s.e.m.	0.50	1.57	1.19	1.41
*p* value	<0.0001	<0.0001	0.0003	<0.0001

Different letters in the same column show significant difference (*p* < 0.05). s.e.m. = standard error of mean. NOR, unaffected PMs; MIL, mild WBM-affected PMs; MOD, moderate WBM-affected PMs; SEV, severe WBM-affected PMs.

## Data Availability

All related data and methods are presented in this paper. Additional inquiries should be addressed to the corresponding author.
